# Enhanced interest in letters and numbers in autistic children

**DOI:** 10.1186/s13229-024-00606-4

**Published:** 2024-06-12

**Authors:** Alexia Ostrolenk, David Gagnon, Mélanie Boisvert, Océane Lemire, Sophie-Catherine Dick, Marie-Pier Côté, Laurent Mottron

**Affiliations:** 1 Département de Psychiatrie et d’Addictologie, Université de Montréal, Montreal, QC H3T 1J4 Canada; 2 Centre de Recherche, Évaluation et Intervention en Autisme (CRÉIA), Rivière-des-Prairies Hospital, CIUSSS du Nord-de-l’île-de-Montréal, 7070 Boulevard Perras, Montreal, QC H1E 1A4 Canada; 3 Autism Alliance of Canada, PO Box 43081, RPO Sheppard Centre, North York, ON M2N 6N1 Canada; 4 St. Michael’s Hospital, Unity Health Toronto, 36 Queen St E, Toronto, ON M5B 1W8 Canada; 5 Département de Psychologie, Université de Montréal, Montreal, QC H3C 3J7 Canada; 6 Département de Psychologie, Université de Sherbrooke, Sherbrooke, QC J1K 2R1 Canada

**Keywords:** Autism, Reading, Hyperlexia, Interests, Language development

## Abstract

**Background:**

An intense and precocious interest in written material, together with a discrepancy between decoding and reading comprehension skills are defining criteria for hyperlexia, which is found in up to 20% of autistic individuals. It may represent the extreme end of a broader interest in written material in autism. This study examines the magnitude and nature of the interest in written material in a large population of autistic and non-autistic children.

**Methods:**

All 701 children (391 autistic, 310 non-autistic) under the age of 7 referred to an autism assessment clinic over a span of 4 years were included. Ordinal logistic regressions assessed the association between diagnosis and the level of interest in letters and numbers. A nested sample of parents of 138 autistic, 99 non-autistic clinical, and 76 typically developing (TD) children completed a detailed questionnaire. Cox proportional hazards models analyzed the age of emergence of these interests. Linear regressions evaluated the association between diagnosis and interest level. The frequency of each behaviour showing interest and competence with letters and numbers were compared.

**Results:**

In the two studies, 22 to 37% of autistic children had an intense or exclusive interest in letters. The odds of having a greater interest in letters was 2.78 times higher for autistic children than for non-autistic clinical children of the same age, and 3.49 times higher for the interest in numbers, even if 76% of autistic children were minimally or non-verbal. The age of emergence of these interests did not differ between autistic and TD children and did not depend on their level of oral language. Non-autistic children showed more interest in letters within a social context.

**Limitations:**

The study holds limitations inherent to the use of a phone questionnaire with caregivers and missing sociodemographic information.

**Conclusions:**

The emergence of the interest of autistic children toward written language is contemporaneous to the moment in their development where they display a strong deficit in oral language. Together with recent demonstrations of non-social development of oral language in some autistic children, precocious and intense interest in written material suggests that language acquisition in autism may follow an alternative developmental pathway.

**Supplementary Information:**

The online version contains supplementary material available at 10.1186/s13229-024-00606-4.

## Background

An intense and precocious interest in written material, together with a discrepancy between advanced decoding and weaker comprehension, has been identified early on in a fraction of children with atypical development [1, 2] and later labeled as hyperlexia [3]. While various levels of discrepancy between reading and understanding abilities have been conceptualized under the broad concept of hyperlexia, this profile of performance and interest has been specifically associated with autism, in which it is observed in 6 to 20% of children depending on the stringency of the criteria applied. In a previous systematic review, we demonstrated that 84% of hyperlexic individuals are on the autism spectrum, which could represent the extreme end of a broader interest and ability toward written material in autism [4].

Typical early language development occurs through social interaction, mostly involving oral productions. A clear indication of a child's engagement in intersubjective communication is demonstrated through either responding to or initiating joint attention [5]. In contrast, a recent systematic review on the relation between joint attention and language established that joint attention does not predict language outcome in a substantial fraction of autistic children [6]. In parallel, reduced reaction to and orientation toward the human voice, is a hallmark early manifestation of autism [7]. This compels us to consider non-relational sources of language exposure as potential contributors to autism development. An enhanced interest in written material could signal an initial development of some language components in a fraction of autistic children.

A recent survey on special interests with parents of autistic children [9 years old on average] found that approximately 30% had a special interest in numbers and in reading [8]. Some basic measures of interest in reading have been developed for typically developing children in the past, but the child’s interest is often assessed based on their enjoyment of shared book reading with an adult [9–11], and not general interest in literacy activities including self-directed ones (with some exceptions, e.g. [12], although their assessment still included social components). There are questionnaires to assess repetitive behaviors or intense interests in autism [13–16], but mostly directed at adolescents and adults, and not specific to reading-related behaviors. They also often focus on impairments and negative impacts of the individual’s interest, such as inflexibility, narrowness, and perseveration on a specific topic.

This study examines the magnitude and nature of the interest in written material in a large population of rigorously diagnosed autistic children and compares them with non-autistic children aged 6 and under. Study 1 evaluates the prevalence and intensity of early interest in letters and numbers in a representative population of children referred for an autism diagnosis assessment in a specific geographic area, comparing the diagnostic reports of autistic individuals to those with other diagnoses; Study 2 replicates Study 1’s questions with a direct parental interview. It further investigates the period of emergence of these interests and their characteristics in autistic children compared to non-autistic clinical children and a typically developing group, and assesses the relationship between the interest in written language and the development of oral language.

## Methods

### Participants

In the Quebec public healthcare system, referrals for an autism diagnosis are made by a general practitioner or pediatrician and confirmed by experienced primary care professionals before the autism diagnosis assessment is performed. There are no severity criteria considered for the referral. The study participants were all the 701 children under age seven referred for an autism diagnosis assessment between 2018 and 2021 at a local public clinic, the CIUSSS-NIM Autism Assessment Clinic (CETSA), where appointments are conducted in French, and a typically developing (TD) group including 76 children from the same geographic and socio-demographic background. The TD children were recruited from 6 daycares in the same geographical area and were required not to have first-degree relatives with a neurodevelopmental condition. The CETSA has exclusive access to preschool age children referred for an autism diagnosis in an administratively defined geographic area in Northern Montreal, Canada with approximately 13,500 births per year. The only exception is children presenting neurodevelopmental symptoms in their first year of life (e.g., major motor delay) who can escape this assessment pipeline because they are referred to another hospital. Our participant sample therefore roughly corresponds to a four-year cumulative incidence of children presenting overt symptoms of autism justifying an assessment without overt, detectable neurogenetic signs.

### Autism assessment process

The autism assessment procedure at the CETSA is performed by one of 13 child psychiatrists or pediatricians along with a psychologist or an educational psychologist, most of whom have 10 to 20 years of experience with autism assessments. Families coming in for an assessment are assigned to clinicians based on availability, and pairs of doctors/professionals are shuffled. The assessment procedure for preschool-age children includes an in-depth interview with the parents in the presence of the child for one to two hours. It is followed by the Autism Diagnostic Observation Schedule (ADOS-2; 17), which can be bypassed either based on the extreme obviousness of the autism diagnosis, or of its absence following the clinical interview and non-standardized observation. An interview with the daycare or school staff is performed when necessary. The diagnosis is established by consensus, based on DSM-V criteria [18]. Clinical certainty is given priority over the child’s ADOS score in case of discrepancy [19].

The *ADOS-2* [17] is a standardized clinician-administrated semi-structured observational assessment. The choice of module to use depends on the child’s communicative language level: the Toddler module is recommended for children 12–30 months who say no words or some isolated words; module 1 is recommended for children over 31 months who say no words or some isolated words; module 2 is recommended for children who can make short sentences at most; module 3 is recommended for children with fluent language.

### Study 1: Assessment reports

*Population (*Table 1*)* Study 1 investigates children’s interest in letters and numbers extracted from the psychiatric or pediatrician assessment reports using a rating grid (described below). It includes all children under the age of 7 assessed at the CETSA during the 4-year period with no exclusion criteria. Among the 701 children, 391 children received an autism diagnosis (the autistic group), and 310 children a negative or alternative diagnosis (the clinical group). A backcheck comparing our participants and the clinic’s list of patients over 2 months of 2018, 2019, and 2020 found that only 2 eligible children referred to the clinic had not been included in the study over these 6 months, because they turned 7 shortly after their assessment and before we had time to contact them.

**Table 1:** Participants demographic information for Study 1: Assessment reports

	Autistic group	Clinical group
	N = 391	N = 310
Sex, n (%)		
Female	96 (25)	52 (17)
Male	295 (75)	258 (83)
Age (months)		
Mean	47.86	57.63
SD	11.51	13.91
Range	24 – 82	18 – 84
ADOS, n (%)		
Toddler module	15(4)	8 (3)
Module 1	282 (72)	105 (34)
Module 2	69 (18)	106 (34)
Module 3	1 (0)	25 (8)
Not assessed with an ADOS	24(6)	66 (21)

To estimate the proportion of autistic children in our geographical area that may have been missed by our combined reference/assessment process in our cohort, we calculated the ratio of the number of autistic children enrolled in our study to the number of children born during this period in the area. Approximately 13,500 children are born every year in the CETSA territory. Our four-year population includes 391 autistic children, resulting in a prevalence of 391/(13,500 × 4) = 0.72%, which is very close to the cumulative incidence of 0.8% at age 7 found in Danish register data [20]. The sex ratio in our autistic participants also matches the reported male-to-female ratio of 4.2 [21].

*Measure–Assessment reports* A rating grid investigating the child’s interest in letters and numbers (Additional file 1) was used to extract relevant information from each child’s psychiatrist or pediatrician assessment report. Reports are concise summaries of the most pertinent information for establishing or ruling out an autism diagnosis. The autism assessment interview does not necessarily incorporate inquiries on this subject, with mention of an interest in the reports occurring only when encountered during the assessment procedure and deemed relevant to the diagnosis. Two raters performed the data collection. When the interest was mentioned, the levels of interest in letters and numbers, respectively, were estimated using an ordinal scale (none < moderate < intense < exclusive). Fifty-six participants’ reports were independently assessed by both raters, resulting in weighted kappa values from 0.86 to 1, indicating a high level of inter-rater agreement. Information about the ADOS module used during the assessment was collected.

*Analyses* The statistical analyses were performed using R software version 4.3.1. [22]. The association between the diagnostic group (autistic or clinical) and the intensity of the interest in letters and numbers, respectively, was evaluated with ordinal logistic regressions. The levels of interest in letters and in numbers (none < moderate < intense < exclusive) were extracted from questions A2 (level of interest in letters) and B2 (level of interest in numbers) of the assessment reports’ rating grid (see Additional file 1). Since 60% of the evaluations were performed by the same child psychiatrist (also the last author of this paper), the models were adjusted for the clinician who performed the evaluation, as an interaction term with the diagnosis. The clinician variable was coded as dichotomic according to if the child had been assessed by the psychiatrist who performed most of the assessments or any other psychiatrist or pediatrician. Models were also adjusted for the age of the child at the assessment. All regressions met the required statistical assumptions. “Brant tests” were used to ensure the respect of the parallel regression assumption of ordinal logistic regressions.

#### Study 2: Caregiver questionnaire

*Population (*Table 2*)* All caregivers of children included in Study 1 were contacted to be offered to participate in Study 2. To be eligible for the study, the caregivers had to be able to answer a questionnaire in French and to know the child since the age of one (excluding late adoptions, for example). If they consented to participate, a phone appointment was made within a month of their child’s assessment for them to answer a caregiver questionnaire (QIMET, described below). 341 caregivers consented to participate and answered the questionnaire on the phone. To be included in the final analyses, the children needed to have a known diagnosis (autistic or non-autistic) or to be TD, a quality score higher than 2/5 as reported by the investigator (see section F of the QIMET), and no missing information about behaviors of interest and competence with letters and numbers. These exclusion criteria resulted in 313 participants being included in the analyses. Children were divided into three groups according to their diagnosis: the autistic group (n = 138), the clinical group (n = 99) and the TD group (n = 76). The most frequently reported alternative diagnoses in the clinical group were language disorder (26.2%) and ADHD (22.2%) (see details in **Table S1 in **Additional file 5**)**.

**Table 2:** Participants demographic information for Study 2: Caregiver questionnaire

	Autistic group	Clinical group	Typically developing group
	N = 138	N = 99	N = 76
Sex, n (%)			
Female	32 (23)	23 (23)	36 (47)
Male	106 (77)	76 (77)	40 (53)
Age (months)			
Mean	51.31	60.78	47.54
SD	11.72	12.62	15.74
Range	24 – 80	30 – 83	24–83
ADOS, n (%)			
Toddler module	4 (3)	0 (0)	N.A
Module 1	112 (81)	27 (27)	N.A
Module 2	16 (12)	37 (37)	N.A
Module 3	1 (1)	5 (5)	N.A
Not assessed with an ADOS	5 (4)	30 (30)	76 (100)

*Measure–Caregiver questionnaire* (in French, *Questionnaire sur l’intérêt pour le matériel écrit chez les tout-petits*; QIMET): The QIMET is a parent reported questionnaire, conducted over the phone, which explores children's interest in letters and numbers, related behaviors and skills, oral language level, parental attitude regarding written material, and relevant qualitative information (Additional file 2). It contains open-ended and close-ended questions, including multiple choice and Likert scale questions. A 5-point quality score on the information gathered during the questionnaire is recorded by the evaluator immediately after the phone interview. Close-ended questions pertaining to the interest or competence with letters and numbers were extracted from the QIMET. To enable comparison and aggregation of items, Likert scale questions were dichotomized: the two highest/strongest response options on one side, and the other options on the other. For example, an item on which the parent had to quantify the portion of their child’s time dedicated to an activity (e.g., question A6 on the QIMET), choosing between "Exclusive (70–100%)", "Majority (45–65%)", "Frequent (25–40%)", "Occasional (10–20%)" and "Absent (0–5%)", was simplified to a binary variable: "Majority" and "Exclusive" were coded as ‘1’ indicating the presence of the sign, and "Absent", "Occasional", "Frequent" were coded as ‘0’ indicating the absence of the sign. Multiple-choice questions and close-ended questions were coded as binary variables according to the presence or absence of the feature.

*Analyses* The ages of emergence of the interest in letters and numbers are presented as Kaplan–Meier plots for each group (autistic, clinical, TD). The association between groups and the time at which the interest emerged was analyzed using Cox proportional hazards models. The autistic group was used as the reference group. Sub-analyses, also using Cox proportional hazards models, were performed on the autistic group only, in order to evaluate whether the age of emergence of the interest was different in the verbal autistic group, compared to the minimally/non-verbal autistic group. ADOS modules were used as a proxy of communicative language levels: autistic children evaluated with ADOS module 2 and up were grouped as language users, and children evaluated with the Toddler module or module 1 were grouped as minimally/non-verbal. Five autistic children (4%) were excluded from this sub-analysis since the ADOS had not been used for their assessment.

The QIMET items were separated into two pairs of categories subsequently to the data collection, but prior to the analyses. Four raters decided if each question was more representative of the child’s interests or competence, and if the behavior described was social, non-social, or irrelevant/ambiguous in this regard. When 3 or 4 raters agreed, the decision was adopted. When the split was equal, the raters discussed until they reached a consensus on the categorization or the exclusion of the question. Five index scores were then computed: (1) non-social interest in letters (e.g., the child likes to align letters), (2) social interest in letters (e.g., the child likes being read to), (3) competence with letters, (4) interest in numbers, and (5) competence with numbers. The score on each index was calculated for each child. The association of the diagnostic group (autistic, clinical, TD) with each index score was quantified using multiple linear regression analyses. The diagnostic group variable was coded as a categorical variable with three categories; the autistic group was used as a reference group in the linear regressions. Each linear regression was also corrected for age. The index scores were standardized within the sample for comparability purposes.

The prevalence of every behavior of interest or competence included in the index scores of the clinical and TD groups were compared to the prevalence in the autistic group using Chi-square analyses to evaluate if some behaviors were overrepresented or underrepresented in the autistic group. P values were adjusted with Bonferroni correction.

## Results

### Study 1: Assessment reports

Demographic information for the full sample is presented in Table 1. Seventy-six percent of the autistic children in our population were minimally verbal or non-verbal, according to the ADOS module used for their assessment (Toddler module or Module 1), and 37% of children in the clinical group. Of note, given the age range of our sample (24–84 months), limited oral language would meet developmental expectations for the children at the lower end of the age range (see **Figure S1 in **Additional File 4 for the age distribution by group and ADOS module).

The intensity of the interest in letters and numbers, respectively, was rated for each child using an ordinal scale (None < Moderate < Intense < Exclusive) according to the clinician reports. Despite the absence of functional communicative oral language in the majority of autistic children, their interest in letters was generally higher than that of the clinical group. Twenty percent of the reports of autistic children mentioned an intense or exclusive interest in letters, when it was the case for only 3% of the clinical group. For autistic children, the odds of having a greater level of interest in letters were 2.78 times higher than for children in the clinical group (OR = 2.78, 95% CI [1.55–5.17], *p* = 8.42e−4). Their interest in numbers was also higher, 17% of autistic children had an intense or exclusive interest in numbers, while it was the case for only 2% of the clinical group. Autistic children had 3.49 times higher odds of having a greater level of interest in numbers than children in the clinical group (OR = 3.49, 95% CI [1.85–6.96], *p* = 2.07e−04). The analyses were adjusted for age and for the clinicians who performed the autism assessment (see **Table S2 in **Additional file 5).

### Study 2: Caregiver questionnaire

Demographic information for the sub-sample used in Study 2 is presented in Table 2.

*Language development* Language level was documented through the choice of ADOS module at the time of assessment and by section C of the QIMET. Eighty-four percent of the autistic children were non-verbal or minimally verbal according to the ADOS module used for their assessment (Toddler module or Module 1), 27% of the clinical group, and none in the typically developing group (no developmental delay in this group). Figure S2 in Additional File 4 shows the age distribution by group and ADOS module. On the QIMET, thirty-three autistic children, 2 in the clinical group, and none in the TD group were reported as completely non-verbal by their caregiver (see Table 3 for detailed results on language ability extracted from items C3, C4, and C5 of the QIMET). Of the 33 autistic children reported as fully non-verbal, the QIMET revealed that 8 could sing the alphabet song, 9 could name letters, 9 could count numbers, 1 could spell some words, 3 did some pretend-reading, and 2 could read some words.

**Table 3:** The most complex oral language level attained by children as reported by caregivers

	Absence of oral language	Isolated words only	Sentence of 3 words	Sentence with conjugated verb
Autistic group	33 (24%)	51 (37%)	18 (13%)	36 (26%)
Clinical group	2 (2%)	6 (6%)	23 (23%)	68 (69%)
TD group	0 (0%)	2 (3%)	4 (5%)	70 (92%)

The table indicates the number of children in each language category, followed by the percentage of the group, rounded up to the nearest whole number

*Presence and intensity of the interest in letters and numbers* Most autistic, clinical, and TD children in our sample had developed at least a moderate interest in letters (autistic: 82%; clinical: 87%; TD: 95%) and numbers (autistic: 83%; clinical: 85%; TD: 97%) at the time of the study. Thirty-seven percent of autistic children had an intense or exclusive interest in letters, 23% of the clinical group, and 25% of the TD group, according to their parents. Thirty-six percent of autistic children had an intense or exclusive interest in numbers, 23% of the clinical group, and 24% of the TD group.

**Fig. 1:**
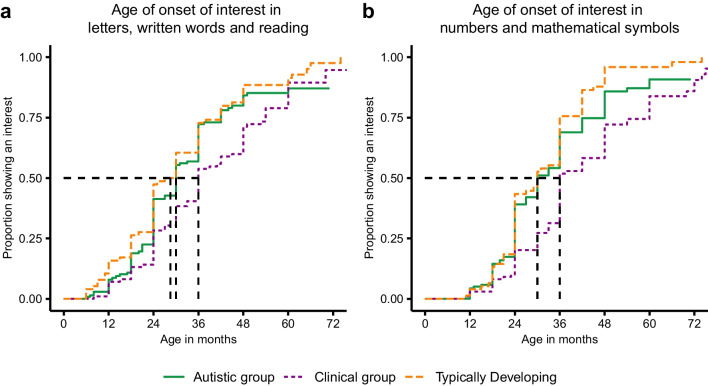
Emergence of the interest in letters and in numbers by diagnostic group. Proportion of autistic, non-autistic clinical, and typically developing children by age of onset of their interest (in months). **a.** Proportion showing an interest in letters, written words and reading by age. **b.** Proportion showing an interest in numbers and mathematical symbols by age

*Age of emergence of the interest in letters and numbers* The median age of emergence of the interest in letters, written words and reading in autistic children was 30 months. Compared to autistic children, the emergence of this interest was delayed in the clinical group (median: 36 months; HR = 0.73, 95% CI [0.55–0.97], *p* = 0.029), but the TD children were no different (median: 28.5 months; HR = 1.17, 95% CI [0.87–1.58], *p* = 0.294) (Fig. 1a). The median age of emergence of the interest in numbers and mathematical symbols in autistic children was 30 months. Compared to autistic children, the emergence of this interest was delayed in the clinical group (median: 36 months; HR = 0.63, 95% CI [0.47–0.84], *p* = 0.002), but the TD children were no different (medians: 30 months; HR = 1.30, 95% CI [0.97–1.75], *p* = 0.077) (Fig. 1b). The age of emergence of both these interests in autistic children did not differ according to the child’s communicative language ability, i.e., between autistic children evaluated with ADOS module 2 and up (grouped as communicative language users) and others evaluated with Toddler module and module 1 (grouped as minimally or non-verbal) (Letters: HR = 0.88, 95% CI[0.51–1.51], *p* = 0.634; Numbers: HR = 0.85, 95% CI[0.49–1.47], *p* = 0.559).

**Fig. 2:**
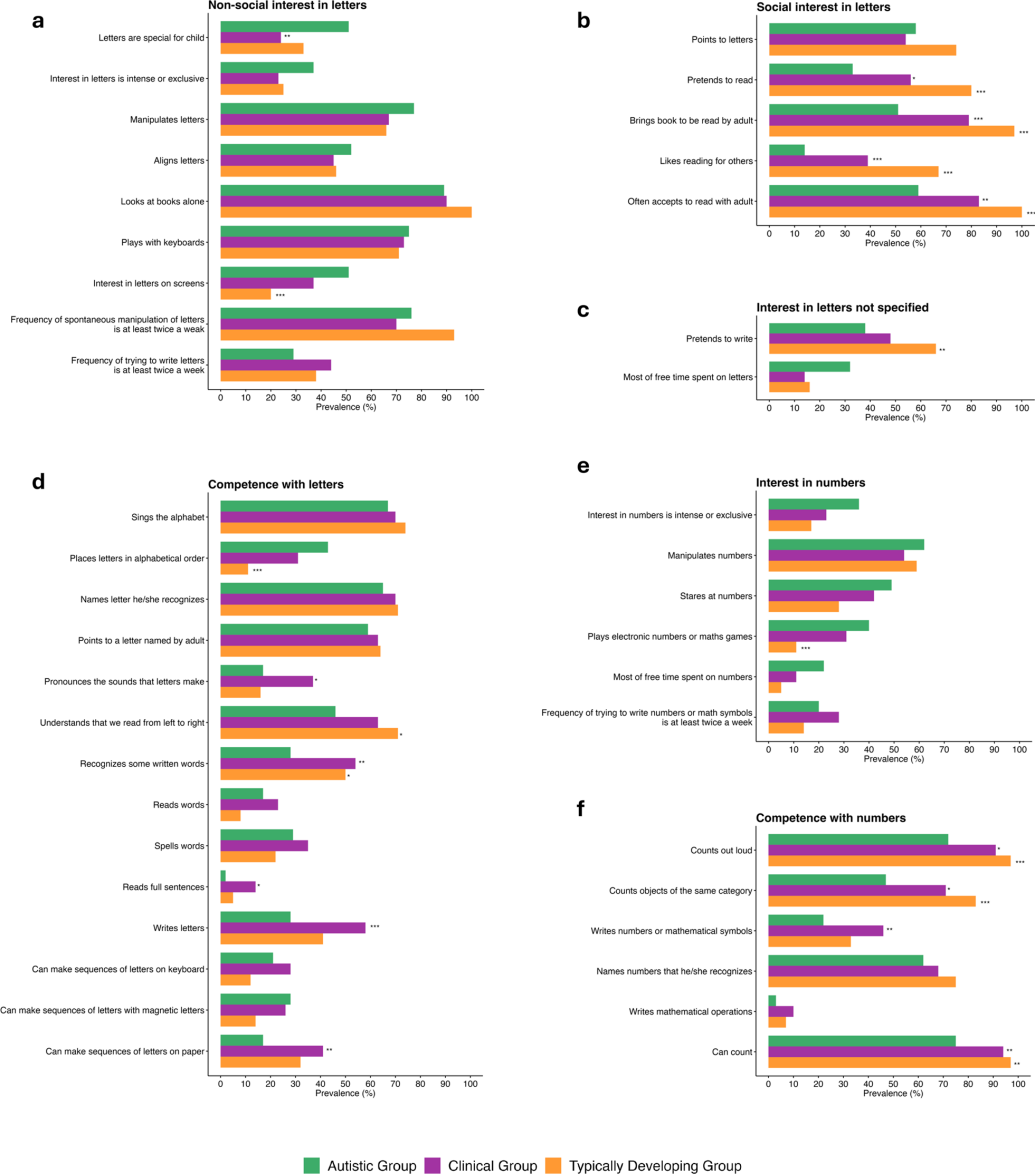
Comparative prevalence of letter- and number-related behaviors of interest and competence grouped under index scores: **a.** non-social interest in letters; **b.** social interest in letters; **c.** interest in letters not specified; **d.** competence with letters; **e.** interest in numbers; and **f.** competence with numbers. Statistical differences are indicated next to the clinical and TD groups showing significant differences compared to the autistic group, with significance levels marked as * (*p* < .05), ** (*p* < .01), and *** (*p* < .001) after Bonferroni correction for multiple comparisons

*Behaviors relevant to the interest in letters and numbers* A detailed questionnaire was used to assess the presence or absence of specific behaviors of interest and competence relating to letters and numbers. Behaviors of interest and competence were grouped under index scores. The prevalence of each behavior of interest and competence within index scores in clinical and TD children, shown in Fig. 2 (see Table S3 for detailed results), were compared to autistic children. The autistic and TD groups share an equivalent age distribution, while the clinical group is slightly older, resulting in a conservative bias in these analyses, if the autistic group shows a higher frequency of these behaviors than the clinical group in spite of being younger on average. Autistic children stand out from clinical children by the fact that letters were “special” for 51% of them (*p* = 0.002). Four out of five behaviors of interest in written material related to a form of social reciprocity were less represented in autistic than clinical and TD children (i.e., pretends to read; brings book to be read by adult; likes reading for others; often accepts to read with adult). Autistic children showed lower levels of competence than clinical and TD children in recognizing some written words, counting out loud, counting objects of the same category and being able to count. Some behaviors of interest and competence were more represented in autistic than TD children, such as interest in letters on screens, the tendency to place letters in alphabetical order, and interest in games with numbers or math. However, these behaviors were no different in frequency from those observed in the clinical group. Similarly, some behaviors of interest and competence were less represented in autistic than clinical children (pronounces the sounds that letters make, writes numbers or mathematical symbols) and TD children (pretends to write, understands that we read from left to right). Some of these differences may be attributed to age, which was not controlled for in these comparisons. The significance level was set at *p* = 0.05 after Bonferroni correction.

*Index scores of interest and competence* When controlling for age, not being autistic was associated with a higher index score for social interest in letters, a particularly large difference in the case of TD children; (clinical: ß = 0.46, 95% CI [0.23–0.70], *p* = 1.0e−04; TD: ß = 1.30, 95% CI [1.05–1.54], *p* = 2.2 e−22). There was an opposite trend for non-social interest in letters, where the clinical group tended to score lower than the autistic group, and the TD group showed no difference (clinical: ß = − 0.41, 95% CI [− 0.68–− 0.14], *p* = 0.003; TD: ß = − 0.16, 95% CI [− 0.43–0.12], *p* = 0.27). Being TD was associated with a higher competence score with letters (clinical: ß = − 0.004, 95% CI [− 0.23–0.22], *p* = 0.98; TD: ß = 0.24, 95% CI [0.01–0.48], *p* = 0.04). Not being autistic was associated with a lower level of interest in numbers (clinical: ß = − 0.384, 95% CI [− 0.64–− 0.12], *p* = 0.004; TD: ß = − 0.537. 95% CI [− 0.81–− 0.27], *p* = 1.2 e−04), despite a generally higher level of competence (clinical: ß = 0.329, 95% CI [0.09–0.57], *p* = 0.007; TD: ß = 0.774. 95% CI [0.52–1.02], *p* = 3.1 e−09). Index scores were standardized within the sample to simplify effect sizes interpretation. Analyses were adjusted for age (See Table 4 and Table S4 in Additional file 5).

**Table 4:** Effects of diagnostic group on standardized index scores of interest and competence, compared to autistic children

	Clinical group			Typically developing group		
	ß	95% CI	*p*-value	ß	95% CI	*p*-value
Non-social interest in letters	− 0.41	[− 0.68–− 0.14]	.003	− 0.16	[− 0.43–0.12]	.27
Social interest in letters	0.46	[0.23–0.70]	1.0 e−04	1.30	[1.05–1.54]	2.2 e−22
Non-social competence with letters	− 0.004	[− 0.23–0.22]	.98	0.24	[0.01–0.48]	.04
Interest in numbers	− 0.38	[− 0.64–− 0.12]	.004	− 0.54	[− 0.81–− 0.27]	1.2 e−04
Competence with numbers	0.33	[0.09–0.57]	.007	0.77	[0.52–1.02]	3.1 e−09

*Notable additional information*: Very few children across groups could read full sentences, but the three participants who could in the autistic group had read their first word at 24, 30 and 36 months, about two years before the 14 participants in the clinical group (mean: 56.5 months; minimum age: 30 months) and the four in the TD group (mean: 51 months; minimum age: 36 months). Several of the autistic children interested in written material displayed “unexpected bilingualism” [23, 24], i.e., they had reached various levels of self-taught skills in a language not spoken in their environment, sometimes to the point of refusing to speak the language most present in their environment, and insisting on speaking English even when their parents could not understand. There were multiple mentions by caregivers of a strong interest in foreign languages and alphabets (e.g., changing the language of video subtitles). The caregivers mentioned the use of tablets, videogames, TV, and YouTube as potential explanations for their child’s foreign language acquisition.

## Discussion

This study investigated the level of interest in letters and numbers in a large, representative population of children who were assessed for autism. This population included children who received an autism diagnosis and others who received an alternative diagnosis. A sub-study within a nested sample, adding a typically developing group of similar age and geographical background, allowed for a more in-depth analysis of these interests.

*Enhanced interest in letters and numbers in autistic children*. Both clinical reports and parental observations establish a higher level of interest in letters and numbers in autistic children as a group. Autistic children have about three times more odds of having a higher interest in letters than children in the clinical group when adjusted for age. According to their assessment reports, 20% of autistic children had an intense or exclusive interest in letters, compared to 3% of the clinical group. This number rose to 37% in the autistic group when caregivers were asked directly about the interest. This prevalence is higher than that reported for hyperlexia in autism, even when broadly defined [4, 25], but similar to a recent study reporting that 29.7% of 9-year-old autistic children had a special interest in reading [8], suggesting the persistence and evolution of hyperlexic features into childhood.

Although 25% of the parents of TD children reported an intense or exclusive interest in letters, this interest did not manifest itself in the same way. It usually started when the children’s oral language was already well developed and focused more on books and shared reading than on letters or other written symbols. Conversely, autistic children engaged in literacy-related activities by themselves and often did not accept external intervention, which may have led parents to underestimate their interest and competence, since they are not displayed in front of them. Other anecdotal behaviors were only ever reported in autistic children, such as compulsive behaviors related to letters and emotional attachment to specific letters and numbers. The interest in letters seems frequent in TD children, although not as frequent as in autistic children, but may be of a very different nature.

*Convergence and differences between Study 1 and 2.* While Study 1 investigates children's interest in letters and numbers extracted from clinical assessment reports in a large representative population, Study 2 delves into parents' perspectives and observations on specific behaviors. The results from Study 1 indicate a lower frequency of intense interests in letters and numbers compared to the results of Study 2, when parents are specifically questioned on this topic. This result was expected, since questions about the interest in letters and numbers are not routinely asked during the autism assessment interview, and clinical reports are only brief summaries of hours of assessment, mentioning the most relevant information according to the current diagnostic criteria. The absence of mention of an interest in the report does not mean that it is not present, as confirmed by the findings of Study 2. Still, an intense or exclusive interest in letters was mentioned for 1 in 5 autistic children in Study 1, while it was very rarely observed in the reports of the clinical group, which attests to the salience of this behavior in autistic children.

*Interest in letters and language development.* While communicative language development is typically delayed in autism [26], the development of an interest in letters and numbers in autistic children as a group does not differ from typically developing children. In a fraction of autistic children, the intensity of the interest in letters surpasses that of typical children, and can result in the very early emergence of reading skills. This interest develops regardless of the children’s level of oral communicative language. The oral communicative language development of our sample was heterogeneous and generally delayed in the autistic group, as indicated by the predominance of ADOS Module 1 in the autistic group, and an equal split between ADOS Module 1 and 2 in the clinical group. All TD children had age-appropriate verbal skills. Although 30 autistic children were reported as completely non-verbal by their caregivers, approximately a third of them named letters and/or numbers, which the caregiver did not consider as words. While the caregivers may have interpreted our question as targeting only communicative language, it may conversely suggest that the autistic children’s emergent literacy skills are “at risk of being underestimated” [27, 28], as is fluid intelligence in prototypical autistic individuals. The presence of a higher level of interest in letters in autism compared to a non-autistic clinical population, even in a sample where 76% of the children present very limited speech, is a novel information that should contribute to the clinical recognition of autism.

The fact that most autistic participants in this study were minimally verbal was expected, given that autism involves a delay in speech acquisition in a substantial proportion of cases [29, 30]. Eventually, speech resumes in most cases and quickly catches up in the school years, drawing a “bayonet-shaped” curve of development [26, 31]. We have suggested that the early phases of the language plateau period manifest a developmental bifurcation towards the treatment of non-socially biased information in autism, such as letters and numbers [32]. In this context, an interest in language-relevant material such as letters and written text could contribute to the future development of special abilities such as early reading ability, and oral language [33, 34]. Early interest in written material could be a precursor for the development of early mathematical and literacy skills, which are important predictors of academic achievement [11, 12, 35].

*Non-social learning of language in autism.* Study 2 revealed that the nature of autistic children’s interest in letters is predominantly non-social compared to non-autistic clinical and typically developing children. This finding converges with the recent finding of independence between language development and joint-attention abilities in studies controlling for the stringency of autistic phenotype and non-verbal IQ [6]. While the absence of overt manifestations of interest in the parents’ oral language is a flagship of an autistic regression in the second year of life, we found that letters were considered “special” for more than half of autistic children according to parental reports, suggesting a preserved interest in linguistic material. There is evidence that hyperlexic abilities can be used to help the development of oral language [36–40] as well as reports of fully formed speech emerging shortly after the onset of reading [41–43]. The anecdotal evidence of non-social acquisition of foreign languages and alphabets in minimally verbal children in the present study adds to this body of evidence. While typically developing children need to acquire oral language abilities prior to reading through explicit teaching [44], some children display an atypical sequence of language development, and learn to decode written text before they can use oral communicative language. Interests in autism can be a learning tool [45]. There is theoretical [46] and empirical [47–49] preliminary evidence that their use in intervention is effective. Strengths and special interests also play a role in autistic people’s wellbeing and quality of life [15, 50, 51]. How the interest in written materials that we have evidenced in autism may become a component of the most established intervention strategies remains to be established [52, 53].

## Limitations

Our study has some limitations. The population under study does not include families who seek an autism diagnostic assessment in the private sector, which is uncommon in our low-income area. In Study 2, we encountered issues with foreign accents, poor sound quality, and distracted responders, but our quality score ensured a minimal standard in the quality of the data. Moreover, caregivers' responses on age-related questions pooled around the year and half-year marks, and they sometimes struggled to recall retroactive information, especially precise ages of onset for specific behaviors. The clinical group was older than the other groups, which could have an impact on data that are highly age-relevant, although we have corrected our analyses for age when possible. In Study 1, we corrected for the clinicians who performed the autism assessment to avoid observational bias, as the senior author of this study assessed 59.9% of the children. Study 2 was not influenced by clinician evaluation, largely eliminating this bias. The raters in Study 1 were not blind to the objectives of the study and participants' diagnosis, but relying on assessment reports limited the possibility of bias in their data entry. We used the ADOS module as a proxy for communicative language level, offering only a rough approximation of the child's level of communicative language at the time of their assessment. Additional information on the children’s language level could only be obtained in Study 2’s questionnaire. Finally, we did not collect or have access to certain information, such as the children’s cognitive level, some sociodemographic parameters, race, and parental education, which could have influenced reading practices and language levels. However, all our families lived in the same geographical area, reducing the risk of large sociodemographic discrepancy.

## Conclusion

Despite a significant delay in oral language development, over a third of autistic children develop an intense interest in letters and numbers, following the developmental timeline of typically developing children. The nature of this intense interest differs from that of non-autistic children, which is predominantly socially oriented. This intense interest may go unnoticed during the diagnostic assessment if parents are not explicitly asked about it. This represents a loss of valuable information for diagnosis and a missed opportunity to explore potential avenues for nonverbal communication, keeping in mind that in a preschool-aged autistic child, not speaking does not necessarily mean not having access to language in another form. Given the positive impact of incorporating children's interests into intervention strategies [47–49] and their significance for the well-being of autistic individuals [15, 50, 51], these results should be considered when evaluating children for autism and designing strengths-based intervention programs.

### Supplementary Information

Additional file 1.

Additional file 2.

Additional file 3.

Additional file 4.

Additional file 5.

## Data Availability

The data collected for the current study cannot be made publicly available due to the detailed behavioral descriptions and medical information that could allow for participants’ identification.

## Abbreviations

ADOS: Autism Diagnostic Observation Schedule
CETSA: CIUSSS-NIM Autism Assessment Clinic
DSM-V: Diagnostic and Statistical Manual of Mental Disorders—5th edition
QIMET: Questionnaire sur l’intérêt pour le matériel écrit chez les tout-petits
TD: Typically developing
