# Supplementary material for: Enhanced interest in letters and numbers in autistic children
Source: Mol Autism. 2024 Jun 12;15:26. doi: 10.1186/s13229-024-00606-4 (PMC11170776; doi:10.1186/s13229-024-00606-4)
Supplement: Supplementary file 1 — Additional file 1. [file 13229_2024_606_MOESM1_ESM.pdf]

A. LES LETTRES

A1. Intérêt spécial qu'a (ou a eu) l'enfant pour les lettres selon le dossier (moment et façon dont cet intérêt s'est manifesté et si l'intérêt a diminué, âge auquel l'intérêt a diminué) :
Si aucun intérêt spécial n'est mentionné au dossier, passer à la question 2.

A2. Niveau d'intérêt pour les lettres :

☐ Aucun ☐ Modéré ☐ Intense ☐ Exclusif

A3. L'enfant présente d'autres intérêts intenses qui sont mentionnés dans le dossier :

☐ Oui ☐ Non

Si oui, lesquels : _________________

B. LES CHIFFRES

B1. Le dossier de l'enfant mentionne un intérêt particulier pour les chiffres :

☐ Oui ☐ Non

B2. Niveau d'intérêt pour les chiffres :

☐ Aucun ☐ Modéré ☐ Intense ☐ Exclusif

C. LANGAGE ORAL

C1. Niveau de langage oral de l'enfant au moment du rapport:

☐ Non-verbal
☐ Limité (quelques mots)
☐ Modéré (combinaisons de quelques mots)
☐ Élaboré (phrases)

C2. Module de l'ADOS qui a été utilisé pour l'évaluation :_________________

C3. L’enfant démontré ou a démontré une prise du poignet : ☐ Oui ☐ Non
Âge d’apparition : _____

C4. L’enfant démontre ou a démontré une régression langagière : ☐ Oui ☐ Non
Âge d’apparition : _____

L’enfant énonce :

- **C5. Des mots inventés** ☐ Oui ☐ Non Âge : _____
- **C6. Du langage répétitif/stéréotypé** ☐ Oui ☐ Non Âge : _____
- **C7. De l’écholalie** ☐ Oui ☐ Non Âge : _____

D. Autres commentaires:

E. Question à l’attention de l’évaluateur du dossier :

L’information contenue dans le dossier semble être:

☐ **Très détaillée** ☐ **Moyennement détaillée** ☐ **Très peu détaillée**
