# Supplementary material for: Enhanced interest in letters and numbers in autistic children
Source: Mol Autism. 2024 Jun 12;15:26. doi: 10.1186/s13229-024-00606-4 (PMC11170776; doi:10.1186/s13229-024-00606-4)
Supplement: Supplementary file 2 — Additional file 2. [file 13229_2024_606_MOESM2_ESM.pdf]

Questionnaire sur l'intérêt pour le matériel écrit chez les tout-petits

<u>Instructions à l'évaluateur/trice, à lire avant la rencontre :</u>

- Ce questionnaire est complété par l'évaluateur d'après les réponses données par le parent, après l'évaluation clinique. Il doit être enregistré et chronométré.

- L'adulte qui répond au questionnaire doit avoir côtoyé l'enfant régulièrement au moins depuis l'âge d'un an (cas des parents adoptifs, grands-parents).

- Les parents qui ont besoin d'un interprète pour comprendre ce questionnaire sont exclus de l'étude mais leur nombre doit être pris en note.

- Avant le premier passage, regarder le questionnaire en entier. Il contient des indications sur la formulation des questions.

- Rappeler au parent qu'il ne doit pas vous révéler le diagnostic s'ils le connaissent, pour éviter tout biais lié à la connaissance du diagnostic lors de la passation. L'information sur le diagnostic pourra être obtenue dans le dossier de l'enfant plus tard et ajouté sur la première page.

- Écrire « NSP » dans les cases si le parent ne sait pas du tout répondre à la question.

- Si les deux parents sont présents et répondent aux questions, le noter. La moyenne de leurs deux réponses pour les âges de début sera notée. Si le parent donne un âge en années, convertir en mois après la passation.

- Avant la passation, mentionner au parent qu'il est important que les réponses soient les plus objectives possibles et basées sur des faits.

- Essayer d'obtenir les réponses demandées et couper le parent s'il apporte des éléments qui ne sont pas pertinents à la question posée. Si les informations ajoutées sont intéressantes, notez sur la dernière page et revenir dessus pour élaborer à la fin. Prévenir le parent pour éviter qu'il ne se vexe « Il m'arrivera de vous couper pendant le questionnaire, mais on pourra revenir sur vos idées à la fin. »

- La page 9 permet d'explorer plus librement tout sujet additionnel ou anecdote intéressante, à l'appréciation de l'évaluateur. Les notes peuvent se prolonger au dos s'il manque de place.

- Remplir la page 10 juste après la passation pour donner vos impressions sur la qualité des réponses recueillies.

© Alexia Ostrolenk, Alina Vocila, Laurent Mottron.

Questionnaire sur l'intérêt pour le matériel écrit chez les tout-petits

Commencer à enregistrer !

Employé/Étudiant présent ☐en personne ou ☐ par téléphone : ______________________

Signature : ______________________

IDENTIFICATION

*Questions qu'on ne pose pas lors de la passation. Les données seront issues du dossier.

Code du participant : ______________________

Sexe : ☐M ☐F ☐Autre

Âge :_________ans et_________mois Date de passation : J J / M M / A A A A

Personne qui remplit le questionnaire et lien avec l'enfant : ______________________

*Diagnostic d'autisme : ☐ Vient d'être posé ☐ En attente d'un diagnostic

☐ Diagnostic alternatif : ______________________

☐ Diagnostic comorbide : ______________________

*OU ☐ Enfant au développement typique

*Autre membre de la famille autiste : ☐ Père ☐Mère ☐ Frère ☐Sœur Autre : _________

Nombre d'enfants TOTAL dans la fratrie : _________

Rang de l'enfant dans la fratrie (1=le plus âgé) : _________

*S'il y a un frère ou une sœur autiste, quel est son rang dans la fratrie : _________

© Alexia Ostrolenk, Alina Vocila, Laurent Mottron.
Questionnaire sur l'intérêt pour le matériel écrit chez les tout-petits (QIMET)

Questionnaire sur l'intérêt pour le matériel écrit chez les tout-petits

A. ÉCRIT : LES LETTRES

A1. Diriez-vous que les lettres sont (ou ont été) spéciales pour votre enfant?

☐ Oui ☐ Non

A2. À quel moment et comment avez-vous remarqué que votre enfant avait un intérêt pour les lettres plus marqué ou précoce que les autres enfants?

A3. Quelle est l'intensité de l'intérêt de l'enfant pour les lettres/les mots écrits/la lecture ?

☐ Aucun ☐ Modéré ☐ Intense ☐ Exclusif

A4. [*Passer si aucun intérêt*] **À quel âge cet intérêt a-t-il débuté? (en mois) :**__________

À quel âge s'est-il arrêté? (en mois, si applicable) : __________

A5. Dans cette liste, quels comportements en rapport avec l'intérêt pour les lettres avez-vous observés chez votre enfant ?

Pour chaque item, dire : **Est-ce qu'il/elle … ? Si oui, à quel âge l'avez-vous vu pour la première fois ? S'il a arrêté de le faire, à quel âge ?**

Quand c'est écrit dans la question : **En quelle langue le fait-il ?** *(entourer anglais et/ou français, écrire langue additionnelle)*

	**Âge de début**	**Âge de fin**
☐ 1. Chante l'alphabet<br>*Anglais/Français/Autre*		
☐ 2. Pointe les lettres autour de lui		
☐ 3. Manipule des lettres (magnétiques/en plastique/en bois/autres)		
☐ 4. Aligne les lettres		
☐ 5. Place les lettres dans l'ordre alphabétique		
☐ 6. Nomme les lettres qu'il reconnait<br>*Anglais/Français/Autre*		
☐ 7. Désigne une lettre si on la nomme		

© Alexia Ostrolenk, Alina Vocila, Laurent Mottron.
Questionnaire sur l'intérêt pour le matériel écrit chez les tout-petits (QIMET)

☐ 8. Prononce les sons que font les lettres (ex.: quand il voit écrit MA, il dit « ma » et pas «èm» et «a»)<br>*Anglais/Français/Autre*		
☐ 9. Comprend qu'on lit/écrit de gauche à droite		
☐ 10. Regarde des livres/des magazines seul/ feuillète/tourne les pages		
☐ 11. Fait semblant de lire pour de faux en émettant des sons différents du texte (jargon, blabla)		
☐ 12. Apporte des livres pour qu'on lui lise		
☐ 13. Aime lire pour les autres (parents, frères et sœurs…)		
☐ 14. Reconnaît des mots écrits		
☐ 15. Lit des mots		
☐ 16. Épelle des mots (liste les lettres qui composent un mot)		
☐ 17. Lit des phrases entières		
☐ 18. Fait semblant d'écrire		
☐ 19. Écrit des lettres : sur papier, dans les airs, sur la tablette….		
☐ 20. Joue avec les claviers d'ordinateurs/téléphone/tablette<br>Outil préféré : ______________________		
☐ 21. S'intéresse aux lettres sur écran, aux sous-titrages de vidéo, génériques de film, bande d'info en continu à la télévision		
☐ 22. Autre activité notable avec les lettres ? À spécifier ______________________		

© Alexia Ostrolenk, Alina Vocila, Laurent Mottron.
Questionnaire sur l'intérêt pour le matériel écrit chez les tout-petits (QIMET)

A6. Quelle quantité de son temps libre est consacrée aux activités en rapport avec les lettres? *Demander un pourcentage et remplir en fonction de la réponse.*

☐ Absent (0-5%) ☐ Occasionnel (10-20%) ☐ Fréquent (25-40%)
☐ Majoritaire (45-65%) ☐ Exclusif (70-100%) ☐ Je ne sais pas

Pour les questions sur la fréquence (L6, L7, L16), poser la question sans proposer les réponses, etcocher la case correspondante. Si ce n'est pas clair ou si le parent hésite, proposer les deux options les plus proches.

A7. Dans les 3 derniers mois, à quelle fréquence est-ce que votre enfant manipule ou observe du matériel lié à la lecture, de sa propre initiative? Par exemple, livre, journal, vidéo sous-titrée, jeux de lettres, …

☐ Jamais ☐ Moins d'1 fois par mois ☐ Moins d'1 fois par semaine
☐ Toutes les semaines ☐ Au moins 2 fois/semaine ☐ Tous les jours

Si l'enfant écrit ou essaye d'écrire : [sinon**, passer à la question 9**]

A8. Dans les 3 derniers mois, à quelle fréquence est-ce que votre enfant essaye d'écrire des lettres?

☐ Jamais ☐ Moins d'1 fois par mois ☐ Moins d'1 fois par semaine
☐ Toutes les semaines ☐ Au moins 2 fois/semaine ☐ Tous les jours

A9. Votre enfant peut-il former des suites de lettres (mots, ou séquences dans l'ordrealphabétique comme « ABCD ») avec … ?

☐ 1. Un clavier (ordi ou téléphone) ☐ 2. Des lettres magnétiques ☐ 3. Sur papier
☐ Je ne sais pas ☐ Aucun
☐ Autres : ____________________

Longueur maximale de la séquence :____________________lettres

A10. Est-ce que l'accès à tablette/téléphone/ordinateur est réglementé chez vous ? Comment ? Quelle est la principale raison ?

A11. Votre enfant a-t-il accès à internet?

☐ Oui ☐ Non ☐ Seulement avec un adulte qui contrôle

© Alexia Ostrolenk, Alina Vocila, Laurent Mottron.
Questionnaire sur l'intérêt pour le matériel écrit chez les tout-petits (QIMET)

Si l'enfant lit des mots ou des phrases : [sinon**, passer à la question 12**]
A12. À quel âge a-t-il lu son premier mot (en mois) ? ________________

A13. Quelle est votre attitude par rapport à la lecture ?

☐ Réticent : je n'aime pas que mon enfant s'intéresse trop aux lettres ou à la lecture
☐ Indifférent : je n'y fais pas attention
☐ Favorable : ça me plait et je l'encourage à jouer avec les lettres ou lire

A14. À quel point est-il important pour vous que votre enfant apprenne à lire maintenant?

☐ Pas important ☐ Minimalement important ☐ Assez important
☐ Très important ☐ Extrêmement important

A15. Quelles activités parmi les suivantes faites-vous avec votre enfant :

☐ Lecture à l'enfant ☐ Enseignement explicite de la lecture/écriture
☐ Guide l'enfant vers matériel écrit ☐ Empêche l'enfant de lire/écrire
☐ Pratique l'alphabet ☐ Pratique les sons des lettres
☐ Autres : ____

A16. À quelle fréquence lisez-vous ou écrivez-vous avec l'enfant?

☐ Jamais ☐ Moins d'1 fois par mois ☐ Moins d'1 fois par semaine
☐ Toutes les semaines ☐ Au moins 2 fois/semaine ☐ Tous les jours

A17. Quel âge avait l'enfant lorsque vous avez commencé à lui lire des livres :

________________ mois

A18. Est-ce que votre enfant accepte qu'on lise avec lui ou pour lui ?

☐ Pas du tout ☐ Le tolère mais n'aime pas cela ☐ Ignore – ne fait pas attention
☐ Souvent ☐ Toujours ☐ Je ne sais pas

A19. Combien de livres sont à la disposition de votre enfant à la maison? Il doit pouvoirles attraper seul.

☐ Moins de 5 livres ☐ 5 à 10 livres ☐ 10 à 20 livres ☐ Plus de 20 livres

A20. Comment pensez-vous que votre enfant a développé cet intérêt et les aptitudes qu'il possède reliées à la lecture, l'écriture, ou le matériel écrit en général?

© Alexia Ostrolenk, Alina Vocila, Laurent Mottron.

B. ÉCRIT : LES CHIFFRES

B1. Quelle est l'intensité de l'intérêt de votre enfant pour les chiffres et les symboles mathématiques ?

☐ Aucun ☐ Modéré ☐ Intense ☐ Exclusif

B2. [Passer si aucun intérêt] **Âge de début de l'intérêt (mois) :** ____________________

À quel âge s'est-il arrêté? (en mois, si applicable) : _________

B3. Dans cette liste, quels comportements en rapport avec l'intérêt pour les chiffres ousymboles mathématiques avez-vous observé chez votre enfant ?

Pour chaque item, dire **: Est-ce qu'il/elle … ? Si oui, à quel âge l'avez-vous vu pour la première fois ? S'il a arrêté de le faire, à quel âge ?**

Quand c'est écrit dans la question **: En quelle langue le fait-il ?** *(entourer anglais et/ou français, écrire langue additionnelle)*

	**Âge de début**	**Âge de fin**
☐ 1. Manipule des chiffres magnétiques/en plastique/en bois		
☐ 2. Compte à haute voix<br>*Anglais/Français/Autre*		
☐ 3. Compte les objets de la même catégorie (marches d'escalier, voitures, formes, etc.)		
☐ 4. Écrit des chiffres ou symboles mathématiques : sur papier, dans les airs, sur les tablettes…		
☐ 5. Nomme les chiffres qu'il reconnaît<br>*Anglais/Français/Autre*		
☐ 6. Fixe les chiffres (numéros de maison, chiffres sur le micro-onde…)		
☐ 7. Écrit des opérations mathématiques (additions, équations, multiplications…)		
☐ 8. Joue sur la tablette/cellulaire/ordi avec des jeux de chiffres / de mathématiques		
☐ 9. Autre activité notable avec les chiffres ? À spécifier ______________________________		

B4. Est-ce que votre enfant sait compter (même un peu)? ☐ Oui ☐ Non
Si oui, jusqu'à quel nombre sait-il compter? ________________

☐ Je ne sais pas

© Alexia Ostrolenk, Alina Vocila, Laurent Mottron.
Questionnaire sur l'intérêt pour le matériel écrit chez les tout-petits (QIMET)

B5. Quelle quantité de son temps libre est consacrée aux activités en rapport avec leschiffres ou les symboles mathématiques?

☐ Absent (0-5%) ☐ Occasionnel (10-20%) ☐ Fréquent (25-40%)
☐ Majoritaire (45-65%) ☐ Exclusif (70-100%) ☐ Je ne sais pas

B6. À quelle fréquence est-ce que votre enfant essaye d'écrire des chiffres ou symboles mathématiques?

☐ Jamais ☐ Moins d'1 fois par mois ☐ Moins d'1 fois par semaine
☐ Toutes les semaines ☐ Au moins 2 fois/semaine ☐ Tous les jours

C. LANGAGE ORAL

Quelles sont les langues parlées ou entendues (si plusieurs langues, indiquer les pourcentages) :

C1. À la maison : ____

C2. À la garderie/ à l'école : ____

[Si l'enfant ne parle pas du tout, **passer directement à la question 12**.]
Veuillez répondre oui ou non aux énoncés suivants :

**L'enfant énonce**	- C**3. seulement des mots isolés**	☐ Oui	☐ Non
	- C**4. des phrases d'au moins 3 mots**	☐ Oui	☐ Non
	- C**5. des phrases avec des verbes conjugués**	☐ Oui	☐ Non
	- C**6. des mots inventés/du jargon**	☐ Oui	☐ Non
	- C**7. du langage répétitif**	☐ Oui	☐ Non
	- C**8. de l'écholalie différée**	☐ Oui	☐ Non

Pour la question C8, définir l'écholalie différée : Vous l'entendez dire une phrase exactementcomme il l'a entendue longtemps avant, de vous, ou de la télévision ou tablette.

**C9. L'enfant répond verbalement quand on lui parle**	☐ Oui	☐ Non
**C10. L'enfant se fait comprendre par le langage oral**	☐ Oui	☐ Non
**C11. L'enfant peut avoir une conversation**	☐ Oui	☐ Non

C12. Quand vous lui donnez une consigne verbale (par exemple, « mets ce papier à la poubelle » ou « enlève tes chaussures »), est-ce qu'il peut l'exécuter ?

☐ Non ☐ Très peu ☐ Parfois ☐ Oui, le plus souvent ☐ Je ne sais pas

© Alexia Ostrolenk, Alina Vocila, Laurent Mottron.

D. JEUX ET LOISIRS

Pour chaque item de la section D, demandez : « est-ce qu'il jouait avec ….? À quel âge? », puis cochez la case correspondante. Proposez les tranches d'âge seulement si le parent n'a aucune idée ou si la réponse n'est pas assez claire pour cocher.

Avec quels jouets ou objets votre enfant s'amusait-il spontanément le plus souvent (mettre une croix dans les cases) :

**Jouet/Objet**	Entre 1-2 ans	Entre 2-3 ans	Entre 3-4 ans	Entre 4-5 ans	Entre 5-6 ans	Maintenant (si plus de 6 ans)
D1. Blocs						
D2. Lego						
D3. Voitures						
D4. Trains						
D5. Autobus						
D6. Avions						
D7. Poupées						
D8. Casse-tête						
D9. Tablette						
D10. Ordinateur						
D11. Téléphone						
D12. Télévision						
D13. Lettres						
D14. Balle/Ballon						
D15. Personnages						
D16. Superhéros						
D17. Animaux						
D18. Peluche/Toutou						
D19. Pâte à modeler - plasticine						
D20. Dinosaures						
D21. Toupies						
D22. Jeux d'encastrement						

© Alexia Ostrolenk, Alina Vocila, Laurent Mottron.
Questionnaire sur l'intérêt pour le matériel écrit chez les tout-petits (QIMET)

D23. Jouets qui font des sons						
D24. Instruments de musique						
D25. À spécifier						

E. AUTRES COMMENTAIRES

L'évaluateur peut explorer librement tout contenu qui lui semble intéressant.

E1. Est-ce que l'enfant a des capacités qui vous étonnent parfois (mémoire, calculs, musique, orthographe ou grammaire, casse-tête, maniement d'applications, habileté motrice, capacité de s'orienter…) ? Si oui, lesquelles et depuis quel âge ?

E2. Y a-t-il d'autres choses intéressantes sur l'intérêt de votre enfant que vous voulez mentionner? Anecdotes, précisions, commentaires…

Fin du questionnaire.

© Alexia Ostrolenk, Alina Vocila, Laurent Mottron.
Questionnaire sur l'intérêt pour le matériel écrit chez les tout-petits (QIMET)

F. Questions à l'attention de l'évaluateur

À remplir immédiatement après la passation.

Temps total de passation_______________ **min**

Sur une échelle de 1 (très faible) à 5 (très bonne) :

F1. Quelle est votre impression sur la précision des réponses du parent ?

Très faible ☐ 1 ☐ 2 ☐ 3 ☐ 4 ☐ 5 Très bonne

F2. Pensez-vous que le parent connaît bien les comportements de l'enfant ?

Très peu ☐ 1 ☐ 2 ☐ 3 ☐ 4 ☐ 5 Très bien

F3. Dans l'ensemble, pensez-vous que les réponses à ce questionnaire sont valides ?

Pas du tout ☐ 1 ☐ 2 ☐ 3 ☐ 4 ☐ 5 Tout à fait

F4. Autres commentaires ?

© Alexia Ostrolenk, Alina Vocila, Laurent Mottron.
Questionnaire sur l'intérêt pour le matériel écrit chez les tout-petits (QIMET)
