# Supplementary material for: Enhanced interest in letters and numbers in autistic children
Source: Mol Autism. 2024 Jun 12;15:26. doi: 10.1186/s13229-024-00606-4 (PMC11170776; doi:10.1186/s13229-024-00606-4)
Supplement: Supplementary file 3 — Additional file 3. [file 13229_2024_606_MOESM3_ESM.docx]

**List of R packages used for the analyses**

For linear regressions and Chi-squared test, we used built-in functions in R version 4.3.1.(R Core Team, 2023). For other analyses we used specific packages.

For built-in library:

- Linear regressions: lm()
- Chi-squared test: chisq.test()

For specific packages:

- Cox proportionate hazards models & Kaplan-Meier analyses:
  - R package "survival" and "survminer" (Kassambara, Kosinski, & Biecek, 2021; Therneau, 2023; Therneau & Grambsch, 2000)
  - Functions : coxph(), survfit()
- Brant test:
  - R package “brant” (Schlegel & Steenbergen, 2020)
  - Function: brant()
- Ordinal logistic regression:
  - R package « ordinal » (Christensen, 2022)
  - Functions : clm()

**Supplementary references:**

Christensen, R. H. B. (2022). ordinal—Regression Models for Ordinal Data. Retrieved from https://CRAN.R-project.org/package=ordinal

Kassambara, A., Kosinski, M., & Biecek, P. (2021). *survminer: Drawing Survival Curves using “ggplot2.”* Retrieved from https://CRAN.R-project.org/package=survminer

R Core Team. (2023). *R: A Language and Environment for Statistical Computing*. Vienna, Austria: R Foundation for Statistical Computing. Retrieved from https://www.R-project.org/

Schlegel, B., & Steenbergen, M. (2020). *brant: Test for Parallel Regression Assumption*. Retrieved from https://CRAN.R-project.org/package=brant

Therneau, T. M. (2023). A Package for Survival Analysis in R. Retrieved from https://CRAN.R-project.org/package=survival

Therneau, T. M., & Grambsch, P. M. (2000). *Modeling survival data: Extending the Cox model*. Statistics for biology and health. New York: Springer.
