# Supplementary material for: Enhanced interest in letters and numbers in autistic children
Source: Mol Autism. 2024 Jun 12;15:26. doi: 10.1186/s13229-024-00606-4 (PMC11170776; doi:10.1186/s13229-024-00606-4)
Supplement: Supplementary file 4 — Additional file 4. [file 13229_2024_606_MOESM4_ESM.docx]

**Figure S1:** Age distribution by group and ADOS module in the entire population of Study 1.

Distribution of ages within the Autistic and Clinical groups based on the Autism Diagnostic Observation Schedule (ADOS) modules used for their assessment.

**Figure S2:** Age distribution by group and ADOS module in the population of Study 2.

Distribution of ages within the Autistic, Clinical, and Typically Developing groups based on the Autism Diagnostic Observation Schedule (ADOS) modules used for their assessment. The data is categorized by group, offering insights into the age distribution within each group across ADOS modules.
