# Supplementary material for: Enhanced interest in letters and numbers in autistic children
Source: Mol Autism. 2024 Jun 12;15:26. doi: 10.1186/s13229-024-00606-4 (PMC11170776; doi:10.1186/s13229-024-00606-4)
Supplement: Supplementary file 5 — Additional file 5. [file 13229_2024_606_MOESM5_ESM.pdf]

Table S1: Alternative diagnoses found in the clinical group

Clinical group diagnoses	n
Other / Not specified	30
Language disorder	26
ADHD symptoms or disorder	22
Developmental disorder	8
Anxiety symptoms or disorder	7
Obsessive-compulsive behaviors or disorder	2
Emotional dysregulation	1
Environmental issues	1
Intellectual disability	1
Selective Mutism	1

Table S2: Effects of autism diagnosis, clinician performing the assessment, and age on the prediction of the level of interest in letters and numbers

	Autism diagnosis		Clinician (another clinician or main investigator)		Diagnosis*Clinician		Age	
	ß	p-value	ß	p-value	ß	p-value	ß	p-value
Interest in letters (ordinal scale: none, moderate, intense, exclusive)	1.02	8.4e-04	0.50	9.6e-02	0.59	1.0e-01	-0.0015	8.1e-01
Interest in numbers (ordinal scale: none, moderate, intense, exclusive)	1.2	2.1e-04	0.70	3.5e-02	-0.0044	9.9e-01	-0.0015	8.2e-01

Note: Odds ratios, not coefficients (ß), are reported in the article.

Table S3: Prevalence of behaviours of interest and competence grouped under index scores

	Autistic group	Clinical group	Adjusted p-value	Typically developing group	Adjusted p-value
n	138	99		76	
**Non-social interest in letters**					
Letters are special for child	70 (51%)	24 (24%)	1.7E-03**	25 (33%)	5.0E-01
Interest in letters is intense or exclusive	51 (37%)	23 (23%)	1.0E+00	19 (25%)	1.0E+00
Manipulates letters	106 (77%)	66 (67%)	1.0E+00	50 (66%)	1.0E+00
Aligns letters	72 (52%)	45 (45%)	1.0E+00	35 (46%)	1.0E+00
Looks at books alone	123 (89%)	89 (90%)	1.0E+00	76 (100%)	1.2E-01
Plays with keyboards	103 (75%)	72 (73%)	1.0E+00	54 (71%)	1.0E+00
Interest in letters on screens	70 (51%)	37 (37%)	1.0E+00	15 (20%)	3.9E-04***
Frequency of spontaneous manipulation of letters is at least twice a week	105 (76%)	69 (70%)	1.0E+00	71 (93%)	6.3E-02
Frequency of trying to write letters is at least twice a week	40 (29%)	44 (44%)	5.9E-01	29 (38%)	1.0E+00

**Social interest in letters**					
Points to letters	80 (58%)	53 (54%)	1.0E+00	56 (74%)	9.4E-01
Pretends to read	46 (33%)	55 (56%)	2.7E-02*	61 (80%)	2.1E-09***
Brings book to be read by adult	70 (51%)	78 (79%)	4.5E-04***	74 (97%)	1.4E-10***
Likes reading for others	20 (14%)	39 (39%)	5.2E-04***	51 (67%)	2.2E-13***
Often accepts to read with adult	82 (59%)	82 (83%)	5.0E-03**	76 (100%)	4.3E-09***
**Interest in letters not specified**					
Pretends to write	52 (38%)	48 (48%)	1.0E+00	50 (66%)	3.4E-03**
Most of free time spent on letters	44 (32%)	14 (14%)	7.3E-02	12 (16%)	4.4E-01
**Competence with letters**					
Sings the alphabet	93 (67%)	69 (70%)	1.0E+00	56 (74%)	1.0E+00
Places letters in alphabetical order	59 (43%)	31 (31%)	1.0E+00	8 (11%)	4.8E-05***
Names letter he/she recognizes	90 (65%)	69 (70%)	1.0E+00	54 (71%)	1.0E+00
Points to a letter named by adult	82 (59%)	62 (63%)	1.0E+00	49 (64%)	1.0E+00
Pronounces the sounds that letters make	24 (17%)	37 (37%)	2.2E-02*	12 (16%)	1.0E+00
Understands that we read from left to right	64 (46%)	62 (63%)	5.6E-01	54 (71%)	2.2E-02*
Recognizes some written words	38 (28%)	53 (54%)	2.1E-03**	38 (50%)	4.3E-02*
Reads words	23 (17%)	23 (23%)	1.0E+00	6 (08%)	1.0E+00
Spells words	40 (29%)	35 (35%)	1.0E+00	17 (22%)	1.0E+00
Reads full sentences	3 (2%)	14 (14%)	1.8E-02*	4 (05%)	1.0E+00
Writes letters	39 (28%)	57 (58%)	2.4E-04***	31 (41%)	1.0E+00
Can make sequences of letters on keyboard	29 (21%)	28 (28%)	1.0E+00	9 (12%)	1.0E+00
Can make sequences of letters with magnetic letters	38 (28%)	26 (26%)	1.0E+00	11 (14%)	1.0E+00
Can make sequences of letters on paper	24 (17%)	41 (41%)	1.8E-03**	24 (32%)	7.2E-01
**Interest in numbers**					
Interest in numbers is intense or exclusive	49 (36%)	23 (23%)	1.0E+00	13 (17%)	1.9E-01
Manipulates numbers	86 (62%)	53 (54%)	1.0E+00	45 (59%)	1.0E+00
Stares at numbers	68 (49%)	42 (42%)	1.0E+00	21 (28%)	8.9E-02
Plays electronic numbers or math games	55 (40%)	31 (31%)	1.0E+00	8 (11%)	2.8E-04***
Most of free time spent on numbers	30 (22%)	11 (11%)	1.0E+00	4 (05%)	6.7E-02
Frequency of trying to write numbers or math symbols is at least twice a week	28 (20%)	28 (28%)	1.0E+00	11 (14%)	1.0E+00

**Competence with numbers**					
Counts out loud	99 (72%)	90 (91%)	1.2E-02*	74 (97%)	2.2E-04***
Counts objects of the same category	65 (47%)	70 (71%)	1.2E-02*	63 (83%)	1.3E-05***
Writes numbers or mathematical symbols	31 (22%)	46 (46%)	4.2E-03**	25 (33%)	1.0E+00
Names numbers that he/she recognizes	86 (62%)	67 (68%)	1.0E+00	57 (75%)	1.0E+00
Writes mathematical operations	4 (03%)	10 (10%)	8.6E-01	5 (07%)	1.0E+00
Can count	104 (75%)	93 (94%)	7.0E-03**	74 (97%)	1.6E-03**

Adjusted p-values by Bonferroni correction are shown.

Table S4: Effects of diagnostic group and age on standardized index scores of interest and competence, compared to autistic children

	Diagnostic group				Age	
	Clinical group		Typically developing group			
	ß	p-value	ß	p-value	ß	p-value
**Non-social interest in letters**	-0.41	2.8e-03	-0.16	2.7e-01	0.013	3.2e-03
**Social interest in letters**	0.46	1.0e-04	1.30	2.2e-22	0.012	1.1e-03
**Non-social competence with letters**	-0.0035	9.8e-01	0.24	4.0e-02	0.042	1.8e-26
**Interest in numbers**	-0.38	3.9e-03	-0.54	1.2e-04	0.015	4.4e-04
**Competence with numbers**	0.33	7.2e-03	0.77	3.09e-09	0.030	2.42e-12
